# The OSE complotype and its clinical potential

**DOI:** 10.3389/fimmu.2022.1010893

**Published:** 2022-09-30

**Authors:** Lejla Alic, Christoph J. Binder, Nikolina Papac-Milicevic

**Affiliations:** ^1^ Department of Medical Biochemistry, Faculty of Medicine, University of Sarajevo, Sarajevo, Bosnia and Herzegovina; ^2^ Department of Laboratory Medicine, Medical University of Vienna, Vienna, Austria

**Keywords:** oxidation-specific epitopes, oxidative stress, DAMPs (damage-associated molecular patterns), complement - immunological terms, natural antibodies (NAbs), pentraxins, immune recognition

## Abstract

Cellular death, aging, and tissue damage trigger inflammation that leads to enzymatic and non-enzymatic lipid peroxidation of polyunsaturated fatty acids present on cellular membranes and lipoproteins. This results in the generation of highly reactive degradation products, such as malondialdehyde (MDA) and 4-hydroxynonenal (4-HNE), that covalently modify free amino groups of proteins and lipids in their vicinity. These newly generated neoepitopes represent a unique set of damage-associated molecular patterns (DAMPs) associated with oxidative stress termed oxidation-specific epitopes (OSEs). OSEs are enriched on oxidized lipoproteins, microvesicles, and dying cells, and can trigger sterile inflammation. Therefore, prompt recognition and removal of OSEs is required to maintain the homeostatic balance. This is partially achieved by various humoral components of the innate immune system, such as natural IgM antibodies, pentraxins and complement components that not only bind OSEs but in some cases modulate their pro-inflammatory potential. Natural IgM antibodies are potent complement activators, and 30% of them recognize OSEs such as oxidized phosphocholine (OxPC-), 4-HNE-, and MDA-epitopes. Furthermore, OxPC-epitopes can bind the complement-activating pentraxin C-reactive protein, while MDA-epitopes are bound by C1q, C3a, complement factor H (CFH), and complement factor H-related proteins 1, 3, 5 (FHR-1, FHR-3, FHR-5). In addition, CFH and FHR-3 are recruited to 2-(ω-carboxyethyl)pyrrole (CEP), and full-length CFH also possesses the ability to attenuate 4-HNE-induced oxidative stress. Consequently, alterations in the innate humoral defense against OSEs predispose to the development of diseases associated with oxidative stress, as shown for the prototypical OSE, MDA-epitopes. In this mini-review, we focus on the mechanisms of the accumulation of OSEs, the pathophysiological consequences, and the interactions between different OSEs and complement components. Additionally, we will discuss the clinical potential of genetic variants in OSE-recognizing complement proteins – the OSE complotype - in the risk estimation of diseases associated with oxidative stress.

## 1 Introduction

### 1.1 Generation of OSE

Increased oxidative stress, characterized by exalted levels of reactive oxygen species, leads to lipid peroxidation of polyunsaturated fatty acids localized in cellular membranes. Lipid peroxidation can be initiated by enzymatic or non-enzymatic mechanisms, resulting in the generation of reactive lipid mediators. Adduction of reactive lipid degradation products to free amino-groups on macromolecules generates novel neo-epitopes, termed oxidation-specific epitopes (OSEs) (1). Once adducted, OSEs have the capacity to alter the function of the affected biomolecule and tag their carrier as “altered-self”. Although there is a large amount of different lipid degradation byproducts that can create OSEs, the best studied examples are malondialdehyde and the more advanced malondialdehydeacetaldehyde, which we are collectively calling MDA (as umbrella term for different MDA-epitopes), 4-hydroxynonenal (4-HNE), 2-(ω-carboxyethyl)-pyrrole (CEP), oxidized cardiolipin (OxCL) and the phosphocholine head group-containing oxidized phospholipids (OxPC).

Within tissues or in the circulation, OSE-decorated structures are considered to be markers of oxidative stress and are found in many pathological conditions such as chronic inflammation, autoimmunity, infections, cancer, and neurological disorders (1–7).

### 1.2 Biological carriers and function of OSEs

The ubiquitous presence of lipids in living cells enables OSEs formation on versatile biological carriers. Major carriers are free biomolecules, oxidized lipoproteins, microvesicles, and apoptotic cells (1).

#### 1.2.1 Free biomolecules

##### 1.2.1.1 Proteins

On proteins, OSEs are attached to the amino groups of lysine and arginine side chains, but modifications of other amino acids have been shown (8–12). This irreversible adduction can alter protein carrier function, lead to aggregation, and increase its immunogenicity (13, 14). The ultimate fate of OSE-modified proteins in homeostasis is proteolytic degradation or clearance by immune responses.

##### 1.2.1.2 Nucleic acids

Nuclear and mitochondrial DNA modifications by MDA and 4-HNE are mutagenic and cancerogenic by causing nucleotide exchange or forming cross-links (11, 15).

##### 1.2.1.3 Phospholipids

Amino group-containing phospholipids, such as phosphatidylethanolamine (PE) and phosphatidylserine (PS), can be modified by MDA, 4-HNE, and CEP (16–21). OSEs on phospholipids alter their recognition by pattern recognition receptors (PRR) and their ability to serve as phospholipase substrates (22–24).

#### 1.2.2 Lipoproteins

##### 1.2.2.1 Low-density lipoprotein

Oxidized LDL has been discovered as the initial carrier of OSEs, where they are considered to be major drivers of atherosclerosis development (25, 26). Following the retention of plasma LDL in the intima of the arterial wall, both the lipid and protein components of LDL can become oxidized (OxLDL). Newly oxidized LDL is pro-inflammatory, chemotactic, and pro-coagulatory (2, 27). Upon OxLDL clearance, engulfing macrophages are converted into foam cells - hallmark cells of atherosclerotic plaques (28–30). Additionally, MDA-, 4-HNE-, OxPC-, and OxCL-epitopes have been documented in atherosclerotic plaques of mice and humans (25, 31–36).

##### 1.2.2.2 High-density lipoprotein

Once modified by OSEs, HDL loses its function to remove cholesterol from cells *via* the ATP-binding cassette transporter A1 (ABCA1) pathway and, *via* CD36, promotes platelet aggregation (37–41).

#### 1.2.3 Microvesicles

Microvesicles are extracellular vesicles (0,1 - 1 µm) with a phospholipid bilayer enriched in phosphatidylserine. They are generated by cellular activation or apoptosis and are pro-coagulatory and pro-inflammatory (2, 42, 43). As carriers of parental cells’ biological cargo, they play a role in inter-organ communication, and alterations in their numbers and content are associated with many pathologies (43, 44). The presence of OSEs, namely MDA- and OxPC-epitopes, has been demonstrated on a subset of circulating MVs and MVs from *in vitro* stimulated cells (45–47).

#### 1.2.4 Dying cells

The presence of OSEs on dying cells and apoptotic blebs seems to be independent of the mechanism of apoptosis induction and the cell types undergoing apoptosis (33, 34, 48–52). Early experiments already proposed that the presence of OSEs on cells undergoing programmed cell death (apoptosis and necrosis) plays a role in their clearance by enhancing their ability to be recognized by phagocytic cells (48, 53).

### 1.3 The function of OSEs as danger-associated molecular pattern molecules

Among well-established DAMPs, like histones, cholesterol crystals, DNA, and others, OSEs represent a distinct group (1, 54). Exposure of OSEs mediates the recognition by innate immunity sensors and can trigger sterile inflammation (51, 52, 55–60). The main cellular innate sensors of OSEs are scavenger receptors and - typically in cooperation - toll-like receptors (TLRs), which are responsible not only for recognition but also for the initiation of downstream signaling events (1).

*In vitro*, treatment with MDA-modified proteins and MDA+ MVs induces cytokine secretion (e.g., interleukin-8 (IL-8), or its murine functional homologues chemokine ligand 1(CXCL1) and CXCL2) in various human or murine cell types and cell lines (47, 51, 52, 61). Moreover, *in vivo*, intravitreal injection of MDA-modified bovine serum albumin (BSA) led to an increase in CXCL1 expression in retinal pigment epithelial (RPE) cells (52). Similarly, in a mouse peritonitis model, injection of MDA-BSA resulted in secretion of CXCL1 and CXCL2 and recruitment of neutrophils and monocytes. Moreover, treatment with the anti-MDA IgM antibody (LR04) attenuated hepatic pro-inflammatory cytokine secretion and leukocyte infiltration induced by the western diet (51). Scavenger receptor A1 (SRA1), CD36, lectin-like OxLDL receptor (LOX1), and CD16 on monocytes and macrophages have been shown to act as sensors for MDA (51, 62–64).

4-HNE increased cytokine secretion, e.g., IL-8, recruitment of neutrophils and macrophages *via* TLR4/NFκB in animal models of atherosclerosis and chronic obstructive pulmonary disease (59, 60). Also, it stimulated the release of pro-coagulatory tissue factor-positive MVs from perivascular cells (65). LOX1 has been identified as the scavenger receptor for 4-HNE (66).

OxPC or OxPC-rich MVs have been reported to induce endothelial cell activation, monocyte recruitment, cytokine secretion (e.g., IL-6) by macrophages, and apoptosis in smooth muscle cells (3, 45, 49, 67). Furthermore, OxPC drives hypercholesterolemia-induced inflammation and atherogenesis and restrains bone formation *in vivo* (4, 35). SRB1, TLR2, and CD36-TLR4-TLR6 heterotrimeric signaling complex are required to recognize Ox-PC (68–70).

CEP has been shown to activate the NLRP3 inflammasome and stimulate the production of IL-1β (71). Moreover, injection of CEP into the mouse eye increased Th1 response and enhanced angiogenesis (72–74). Additionally, in a peritonitis model, CEP generated by neutrophils promotes infiltration of monocytes and macrophages by binding to β2 integrins on their surfaces (75). For CEP binding and clearance, the coordinated action of CD36 and TLR2 is required (76).

## 2 Recognition of OSEs by innate humoral immunity

Both innate and adaptive immune responses against OSEs have been demonstrated, and their functional implications are being elucidated. Thus, in this mini-review we will focus solely on representatives of soluble innate immune responses to OSEs: natural IgM antibodies, pentraxins, and several components of the complement cascade because their levels and genetic variants have been implicated in the development of diseases associated with increased oxidative stress (**Table 1**).

**Table 1:** Plasma proteins recognizing OSEs and their reported biological effects.

Complement component	OSEs	The biological effect of OSE binding	Reference
Natural antibodies	MDA-, OxLDL	Clearance and neutralization of OxLDL-, MV-, and apoptotic cells-induced inflammation; protection against atherosclerosis and CVDs; inhibition of MV-mediated coagulation	(42, 45, 47, 77–92)
CRP	OxPC-	Binding to apoptotic cells and in atherosclerotic lesions; activation of CCC	(49, 93, 94)
PTX3	OxLDL	Promoting OxLDL uptake by macrophages	(95, 96)
C1q	MDA-, OxLDL	CCC activation; clearance of oxLDL in an anti-inflammatory manner	(97–100)
C3a	MDA-	OxLDL facilitates clearance of C3a by macrophages	(101, 102)
CFH	MDA-^a^, OxLDL (OxPC-)^b^, 4-HNE-^c^, CEP-^d^	Decreasing inflammation^a^, inhibition of complement activation^b,d^, protection from cell death^c^	(52, 103–109)
FHR-1	MDA-	Propagation of inflammation and deregulation of CFH function	(105, 110, 111)
FHR-3	MDA-, CEP-	Propagation of inflammation	(105, 112)
FHR-5	MDA-	Reducing CFH cofactor activity and increasing C3 deposition	(113)

The superscript letter in the second column designates the reported biological function in the third column.

### 2.1 Natural antibodies

Natural IgM antibodies are pre-existing antibodies that typically contain unmutated variable regions encoded by germline gene sequences. In mice, natural antibodies are secreted by B1 cells, but marginal zone B cells may also contribute to their production. Natural antibodies arise in newborns without infections or exposure to exogenous antigens and thus can be found in gnotobiotic mice. Thirty percent of all natural IgM antibodies have specificity for OSEs, such as MDA, OxPC, and 4-HNE, among which MDA is the predominant antigen (50). A series of OSE-specific natural IgM antibodies have been cloned, of which the best characterized are LR04, NA17, and E014 (recognizing MDA), T15/E06 (recognizing OxPC), and LR01 (recognizing OxCL) (25, 33, 34, 48, 50, 114). They bind to microbial antigens and altered-self structures, which allows them to mediate important functions in host defense, but also makes them essential in homeostasis maintenance, respectively (115). Natural IgM antibodies neutralize the pro-inflammatory effects of oxidized lipids and MVs, mediate apoptotic cell clearance, and are anti-atherogenic by blocking OxLDL uptake and foam cell formation (45, 47, 77–79). Additionally, we have shown that anti-MDA IgM hinders the binding of coagulation factors X/Xa on MVs, attenuating the propagation of coagulation and protecting from pulmonary thrombosis in mice (42). Moreover, MDA-targeted passive and active immunization strategies that increase the levels of MDA-specific IgM protect from atherosclerosis and hepatic inflammation (80–82). Furthermore, mice unable to secrete natural IgM antibodies display impaired clearance of apoptotic cells and develop arthritis and lupus-like disease, which can be in part explained by the lack of OSE-specific IgMs (83–85). Finally, studies in various human cohorts demonstrated that low levels of IgMs against OSEs are associated with an elevated risk of developing cardiovascular diseases (CVDs), confirming the beneficial role of anti-OSE IgMs (86–92).

### 2.2 Pentraxins

Pentraxins are acute-phase proteins and represent soluble innate pattern recognition proteins. As such, they facilitate the removal of invading microorganisms and damaged host cells. There are two types of pentraxins, short (C-reactive protein (CRP) and serum amyloid P (SAP)) and long ones (pentraxin 3 (PTX3)) (116). CRP has been shown to bind to OxPC on OxLDL, and they colocalize on the surface of apoptotic cells and in human atherosclerotic lesions (49, 93). When CRP is complexed with OxPC-epitopes, it recruits C1q and activates the C1 complex of the classical complement cascade (CCC) (94). In contrast to CRP, SAP and PTX3 do not bind to OSEs, although SAP competes out the binding of plasma IgM and CRP on late apoptotic cells (93). Interestingly, OxLDL enhanced the expression of PTX3, which promoted OxLDL uptake by macrophages and blocked cholesterol efflux (95, 96). Furthermore, PTX3 protects against 4-HNE-induced complement activation by recruiting CFH to the basal RPE and inner Bruch’s membrane in AMD (117).

### 2.3 Complement components

The complement cascade protects and orchestrates the removal of invading pathogens and altered self- or foreign-structures by employing three pathways. Its activity is steered by complement activators and regulators that prevent collateral damage to host tissues.

#### 2.3.1 C1q

C1q is a multimeric protein, a part of the initiator complex of the CCC. With its globular head, it can recognize pathogen-associated molecular patterns (PAMPs), DAMPs, and immune complexes, and once bound, it activates the CCC with the collagen-like domain. It binds to OxLDL, which leads to the complement activation and deposition of C3b, facilitating OxLDL uptake by monocytes and macrophages (97, 98). Additionally, the engulfment of OxLDL with C1q suppresses macrophage NFκB and NLRP3 activation, resulting in an enhancement of IL-10 and a reduction in IL-1β secretion (99). The initial notion that the binding of C1q to oxidized lipoproteins is mediated through OSEs came from the finding that MDA-LDL binds C1q, resulting in the deposition of C4b and activation of the CCC, which can be inhibited by ApoE (100). Furthermore, C1q is found both on circulating MVs, and apoptotic cells; however, if this binding is (in part) OSE-dependent has not been investigated so far (118–120). Due to the impairment of apoptotic cell clearance, C1q deficiency in mice and men predisposes to the development of systemic lupus erythematosus (SLE) (118, 121).

#### 2.3.2 C3a

Complement anaphylatoxin C3a is a small degradation product of C3 generated by C3 convertase. It is a chemotactic molecule of the immune system, and although classically considered a pro-inflammatory molecule, C3a has been shown to have some anti-inflammatory functions (122, 123). MDA-epitopes are ligands for C3a on OxLDL and apoptotic cells. Furthermore, this OxLDL-C3a interaction results in increased internalization of C3a by macrophages, thus making OxLDL a platform enhancing uptake of C3a (101). Myeloperoxidase-rich MVs can also be the carriers of C3a; however, if recruitment of C3a to MVs is mediated by OSEs has to be elucidated (124).

#### 2.3.3 Complement factor H

Complement factor H is the regulator of the alternative complement pathway. It comprises 20 short consensus repeat (SCR) domains and acts as a sensor of PAMPs and DAMPs. Additionally, a splice variant of CFH exists – factor H-like protein 1 (FHL-1). Impairment of CFH functions contributes to the development of many diseases, with AMD and atypical hemolytic uremic syndrome as the most prominent examples (125, 126). Weismann et al. demonstrated that CFH and FHL-1 recognize MDA-epitopes and colocalize within the retina and atherosclerotic lesions. MDA recognition is achieved by SCR7 and SCR19-20, where SCR7 is the most critical, but SCR19-20 also matter (103–106). CFH protects from MDA-induced IL-8 secretion and inactivates C3b into iC3b on MDA-carrying surfaces (52). Furthermore, the CFH variant Tyr402His (rs1061170) within SCR7 that predisposes to AMD results in decreased binding to MDA-epitopes in healthy individuals and AMD patients (52, 105). Similarly, transgenic mice with human SCR6-8 402His inserted into a mouse CFH display an AMD-like phenotype (103). In addition, CFH binds CEP-decorated surfaces and this binding is attenuated in the presence of complement factor H-related protein 3 (FHR-3) (112). The observation that CFH binds to OxPC-epitopes requires further validation (52, 107, 108). Although CFH does not directly interact with 4-HNE-epitopes, it protects ARPE-19 cells from 4-HNE-induced cell death by attenuating apoptotic and necroptotic cell death pathways (52, 109). Next to OSEs, CFH binds many other DAMPs on the surface of dying cells, apoptotic blebs, and MVs (52, 110, 127, 128). There, CFH compensates for the loss of membrane-bound complement inhibitors by protecting cells from excessive complement activation and limiting inflammatory potential (127, 128).

#### 2.3.4 Complement factor H-related proteins

FHRs are five plasma proteins that share high structural and functional similarities with CFH, among other recognition of OSEs by FHR-1, 3-, and -5. Interestingly, FHRs do not have potent complement regulatory activity like CFH. Their competition with CFH in recruitment to various ligands labels them as “deregulators of CFH activity” (129, 130).

Using a genome-wide association study, we identified FHR-1 as the main competitor to CFH for binding MDA-epitopes in a cohort of healthy individuals. Once bound to MDA-epitopes, FHR-1 blocks CFH-mediated C3b inactivation, allowing C3b and Bb deposition and propagation of the alternative complement pathway. MDA-epitopes on necrotic cells are recognized by FHR-1 *via* SCR1-2 (105, 110, 111). This activates monocytes in the vicinity *via* EMR2 receptors and NLRP3 pathway (111). Consequently, in necrotic cores of atherosclerotic lesions, FHR-1 colocalizes with macrophages and stimulates IL-1β and IL-8 secretion (110). Considering its property to bind MDA-epitopes, deletion of the gene encoding for FHR-1 (CFHR1) is assumed to be beneficial in chronic inflammation. Indeed, carriers of this deletion have a reduced risk of atherosclerotic CVDs and anti-neutrophil cytoplasmic antibody-associated vasculitis and display lower levels of inflammatory markers (110, 111). Therefore, on MDA-carrying host surfaces, FHR-1 is pro-inflammatory. Of note, FHR-1 does not bind any other OSEs (105).

The deletion of CFHR3&CFHR1 genes was shown to enhance CFH binding to MDA-epitopes because, as FHR-1, FHR-3 competes for them. Compared to FHR-1 and CFH, it displays the lowest affinity towards MDA-epitopes. Once bound to MDA-epitopes, FHR-3 does not cause deregulation of CFH function (105). Moreover, FHR-3 has been shown to also bind to CEP-epitopes but does not interact with OxPC- and 4-HNE-modifications (105, 112). Interestingly, when attached to the surface of polarized senescent ARPE-19 cells, FHR-3 is internalized. Engulfed FHR-3 drives pro-inflammatory responses of RPE cells by upregulating C3 and factor B expression and translocating newly generated C3a from the cytoplasm to the membrane (112). Although the relation between OSE-binding and FHR-3 has not been investigated in diseases associated with a high level of oxidative stress, in many of them, e.g., rheumatoid arthritis and SLE, FHR-3 serum levels are increased, and the lack of CFHR3 gene is protective (131).

Complement factor H-related protein 5, one of the largest members of the FHR family, attaches itself to apoptotic and necrotic cells through SCR5-7 domains. MDA-epitopes were shown to be the predominant ligands responsible for this recruitment, as demonstrated by the fact that the density of these epitopes determines the amount of FHR-5 bound. Like FHR-1 and FHR-3, when attached to MDA-epitopes, FHR-5 reduces CFH cofactor activity and enhances C3 deposition. Additionally, a hybrid protein FHR-21-2-FHR-5 obtained from the serum of a patient with C3 glomerulopathy binds to MDA-epitopes (113). When adhered to apoptotic and necrotic cells’ surfaces, FHR-5 and FHR-1 recruit CRP and PTX3 and activate classical and alternative complement pathways, facilitating opsonization (132).

### 2.4 The connection between natural IgM antibodies, pentraxins, and complement proteins

Although OSE-recognizing IgM antibodies and pentraxins have been shown to instruct phagocytic cells for clearance of damaged structures individually, they can also employ the complement cascade to ensure even more efficient and potent removal machinery (1, 49, 114, 133).

Natural IgM antibodies, CRP, and SAP have been shown to recruit early components of the CCC, such as C1q, mannose-binding lectin, and ficolins in efferocytosis (49, 84, 93, 134–137). Additionally, on the surface of apoptotic cells, regulators of complement activity - C4-binding protein (C4BP) and CFH - can attach directly to DAMPs or indirectly, e.g., through CRP (108, 138, 139). Recruitment of these complement regulators prevents the assembly of the membrane attack complex and lysis, keeping efferocytosis immunosilent. After engulfment of apoptotic cells opsonized with CRP and complement, macrophages maintain an anti-inflammatory status (136, 140, 141). In contrast to other complement components, FHR-1, -3, and -5 have been shown to bind to the surface of necrotic cells *via* OSEs, CRP, or other DAMPs to enhance opsonization by complement activation and act pro-inflammatory (111, 113, 132, 142).

Importantly, it has to be kept in mind that the combined recognition of MDA and any other co-expose OSE by various humoral immune responses as well as the cellular receptors binding them and/or the cellular receptors binding OSE directly will ultimately determine the net biological effect. The elucidation of these functional responses will provide insights into the pathophysiological relevance of OSE recognition by humoral immunity.

## 3 OSEs, recognition of OSEs and its clinical potential

Structures modified by OSEs have been detected practically everywhere, in various tissues and body fluids (4, 10).

As markers of enhanced oxidative stress, OSEs occur early, contribute to disease development, and have strong biomarker potential that has not been explored enough. The most commonly used assay for lipid peroxidation, thiobarbituric acid reactive substances assay (TBARS), is not specific, while mass spectrometry, immunological, chromatography, and imaging techniques are much more specific and reliable but costly. Still, even today, TBA-based assays are used in many clinical studies (143, 144). In contrast, studies that monitor immune responses against OSEs and their effects have not been broadly performed. Many such investigations have been conducted for atherosclerosis as a prototypical OSE-driven pathology with a vital contribution of the immune system. Evidence obtained from this research supports the observation that IgG antibodies to OxLDL are pro-atherogenic and increase the risk of developing CVDs – though this association may be more complex and depend on the IgG isotype. In contrast, IgM antibodies to OxLDL have largely shown to be associated with atheroprotection (145, 146). An even more precise prediction for CVD events was observed in a prospective 15-year-long study when the multivariable prediction models, including levels of oxidized phospholipids (OxPL)/apolipoprotein B (apoB) as OSEs, OSE-specific IgM and IgG antibodies, and CRP were used (88). It is known that the levels of CRP correlate with the levels of OSEs (MDA and 4-HNE), but if this association depends on CRP genetic variants influencing CRP levels and/or recruitment to damaged surfaces have not been investigated so far (147).

Furthermore, OSE-recognizing complement components have been associated with the development of autoimmunity, emphasizing the importance of these proteins in the clearance of damaged cells and prevention of autoantigen spill-over. So far, the interaction between C1q and MDA has not been characterized; therefore, no polymorphisms in C1q were shown to influence MDA-binding or levels. However, congenital C1q deficiency is associated with the development of lupus-like autoimmunity due to the impaired clearance of apoptotic cells (118, 148). Interestingly, in SLE, the OSE levels (MDA, 4-HNE, and OxPC) and OSE-specific IgM and IgG antibodies are altered (149–151).

The most important modulators of CFH binding to MDA-epitopes – the CFH variant Tyr402His and the deletion of CFHR3&CFHR1 genes – are frequent in the population and affect the development of the two most common diseases, atherosclerosis and AMD (52, 105). Although these diseases affect different organs, they have a similar underlying pathology linked with increased oxidative stress. Deletion of CFHR3&CFHR1 genes offers protection in both of them, while Tyr402His is deleterious in AMD and possibly atherosclerosis, highlighting CFH’s importance in homeostatic responses (110, 152–155). Since CFH is a crucial player in regulating complement activation, variants affecting its activity have been used in combined genetic risk scores/haplotypes for some complementopathies (153).

**Figure 1:**
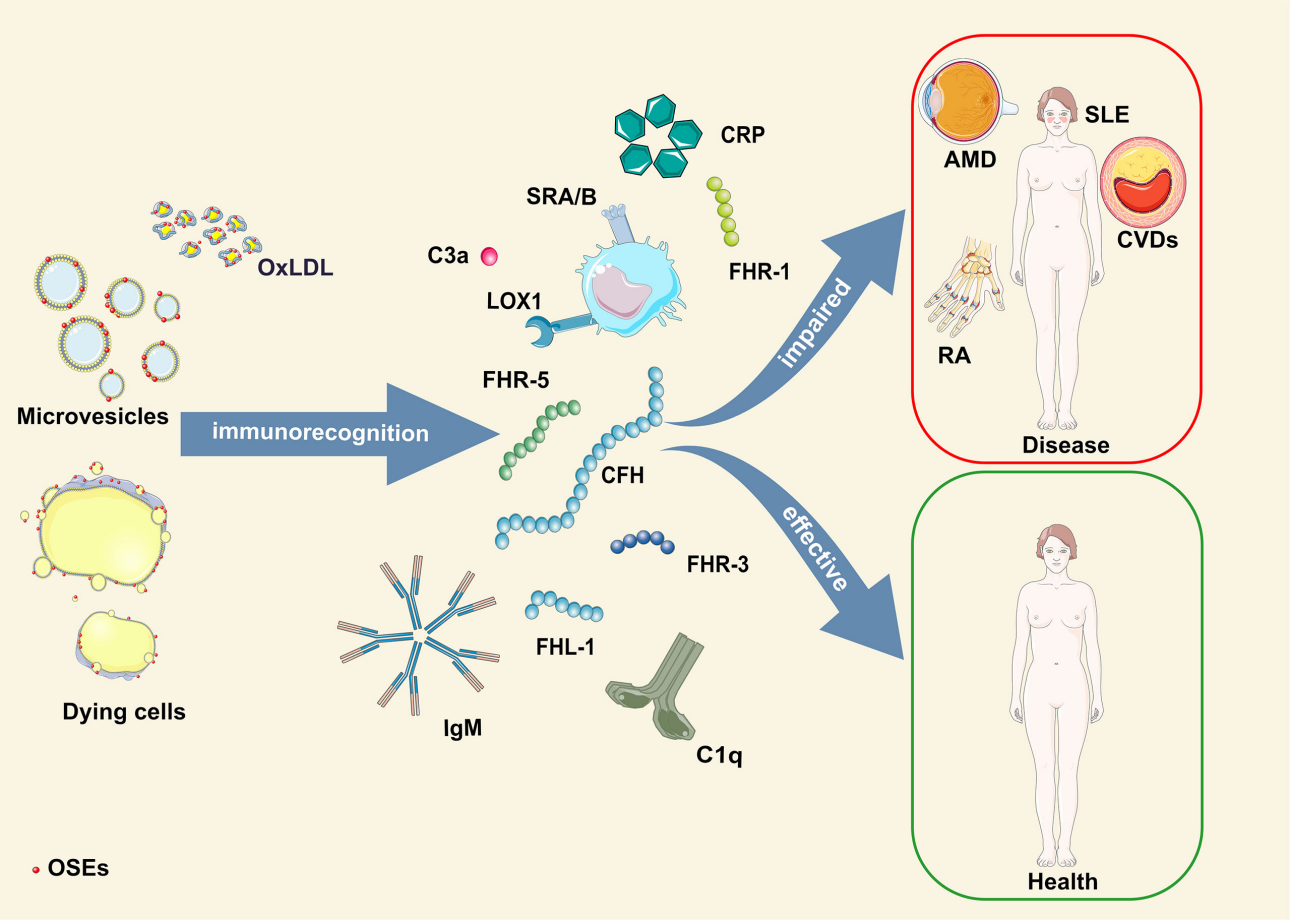
Schematic illustration of OSE humoral immunorecognition and consequences thereof. AMD, age-related macular degeneration; CFH, complement factor H-related protein; CRP, C-reactive protein; CVDs, cardiovascular diseases; FHL-1, factor H-like protein 1; FHR-1, -3, -5, complement factor H-related protein 1, 3, 5; OSE, oxidation-specific epitopes; OxLDL, oxidized low-density lipoprotein; RA, rheumatoid arthritis; SLE, systemic lupus erythematosus.

Based on the available literature, it is evident that the early appearance of OSEs and innate humoral immune responses are critical players in the development and progression of pathologies caused by oxidative stress (**Figure 1**). Therefore, using individual genetic variants or levels of OSE-recognizing proteins in disease prediction models generates less accurate prediction scores. To obtain a more precise, personalized risk prediction score for the ability of the host to deal with increased oxidative stress, we suggest using integrative analysis that considers all individual OSE-related parameters combined (levels of OSE and OSE-recognizing proteins and genetic variant within OSE-recognizing proteins). A strong argument for such an approach comes from a study in which combining CEP levels with AMD risk alleles in ARMS2, HtrA serine peptidase 1 (HTRA1), CFH or C3 showed a twofold to threefold increased risk score compared to a genotype-based score alone (156).

Thus, we propose a novel concept – the OSE complotype. The OSE complotype would include levels of specific OSEs, immune responses involved in their detection and clearance, and a repertoire of inherited genetic variants that modulate properties of OSE-recognizing proteins. This integrative method would allow for a more precision medicine-directed approach to evaluate the individual risk, progression, and therapeutic responses in oxidative stress-related diseases.

## Author contributions

All authors listed have made a substantial, direct, and intellectual contribution to the work and approved it for publication.

## Funding

CJB was supported by grants of the Austrian Science Fund (SFB F54) and the Leducq Foundation (TNE-20CVD03). NPM was supported by the Vienna Science and Technology Fund (WWTF LS20-081).

## Acknowledgments

The figure was created using Servier Medical Art (https://servier.com/en/brochure/servier-medical-art/).

## Conflict of interest

The authors declare that the research was conducted in the absence of any commercial or financial relationships that could be construed as a potential conflict of interest.

## Publisher’s note

All claims expressed in this article are solely those of the authors and do not necessarily represent those of their affiliated organizations, or those of the publisher, the editors and the reviewers. Any product that may be evaluated in this article, or claim that may be made by its manufacturer, is not guaranteed or endorsed by the publisher.
